# Primary malignant melanoma of the female urethra: A rare case report

**DOI:** 10.1016/j.eucr.2025.103133

**Published:** 2025-07-22

**Authors:** Anahita Ansari Djafari, Mohammad Seifi Poor, Sina Samenezhad, Reza Nicknama, Azadeh Rakhshan

**Affiliations:** a Laser Application in Medical Sciences Research Center, Department of Urology, Shahid Beheshti University of Medical Sciences, Tehran, Iran; b Department of Urology, Shohada-e-Tajrish Educational Hospital, School of Medicine, Shahid Beheshti University of Medical Sciences, Tehran, Iran; c Department of Pathology, Shohada-e-Tajrish Educational Hospital, School of Medicine, Shahid Beheshti University of Medical Sciences, Tehran, Iran

**Keywords:** Urethral melanoma, Urethral caruncle, Immunotherapy, Primary malignant melanoma

## Abstract

A 61y/o woman who presented with a dark, bleeding mass at the urethral meatus, accompanied by dysuria and significant weight loss. Initial diagnosis by a GP suggested a urethral caruncle. Imaging revealed a urethral mass, which was surgically excised. Histopathological examination confirmed the diagnosis of malignant melanoma. Despite complete surgical resection, follow-up imaging 6 months later revealed widespread metastases. She was referred for systemic and immunotherapy. This case underscores the importance of considering urethral melanoma in the differential diagnosis of urethral masses, especially when the presentation mimics benign conditions. Early recognition, thorough staging and prompt intervention are crucial for improving outcomes.

## 1 Introduction

Malignant melanoma in the urethra is a rare tumor that is difficult to diagnose and treat, leading to a poor prognosis.^1^ Primary malignant melanoma of the urethra is a rare and aggressive neoplasm that accounts for less than 1 % of all melanomas and approximately 4 % of urethral malignancies.^2^ It predominantly affects females, particularly in the postmenopausal age group, and is often associated with a poor prognosis due to its high recurrence rate and early metastatic potential.^3^ Diagnosis is challenging because urethral melanoma frequently mimics benign conditions such as urethral caruncles or polyps. The clinical presentation is often nonspecific, including bleeding, dysuria, or the presence of a urethral mass. Due to its rarity, there are no standardized treatment protocols, and management typically involves surgical excision followed by systemic therapy in cases of recurrence or metastasis.

In this report, we present a rare case of primary urethral melanoma in a female patient with a prior history of cervical adenocarcinoma, highlighting the diagnostic challenges and discussing therapeutic considerations.

## 2 Case presentation

**Fig. 1:**
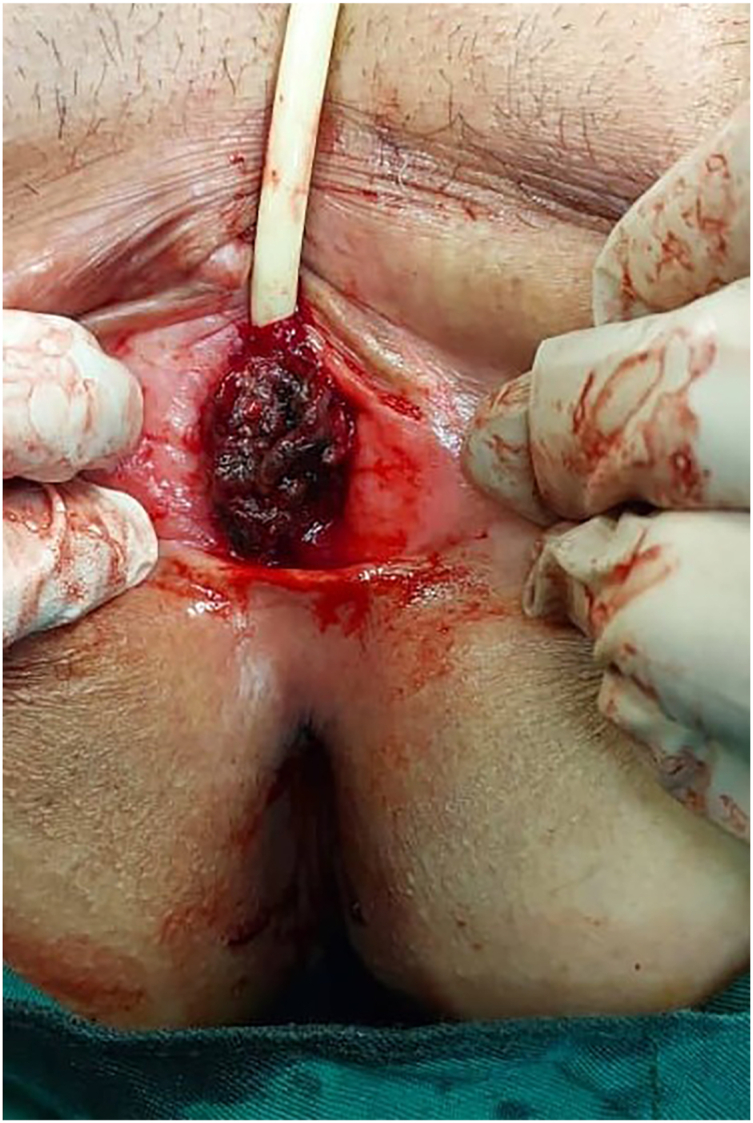
Urethral mass.

A 61-year-old postmenopausal woman presented to our urology outpatient clinic with complaints of dysuria, weight loss, and a dark mass protruding from the urethral meatus and anterior vaginal wall (Fig. 1). The mass had been present for approximately five months and had been previously diagnosed as a urethral caruncle by her general practitioner. Additionally, she reported intermittent bleeding from the urethral meatus.

Her past medical history was significant for stage PT2N0Mx invasive cervical adenocarcinoma, for which she had undergone neoadjuvant chemotherapy followed by total abdominal hysterectomy with bilateral salpingo-oophorectomy (TAH + BSO) and chemoradiotherapy seven years earlier.

On physical examination, a pigmented, pedunculated mass was observed at the urethral meatus, partially obstructing the urethral opening. No cutaneous lesions or discoloration were noted. Abdominal examination was unremarkable, with no organomegaly or palpable masses.

**Fig. 2:**
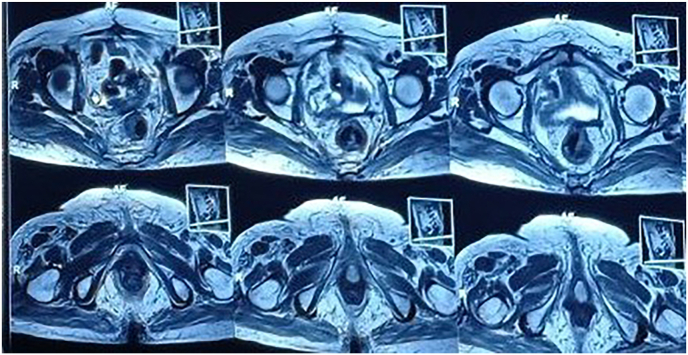
MRI showing an enhancing lesion in anterior aspect of the distal vaginal cuff.

**Fig. 3:**
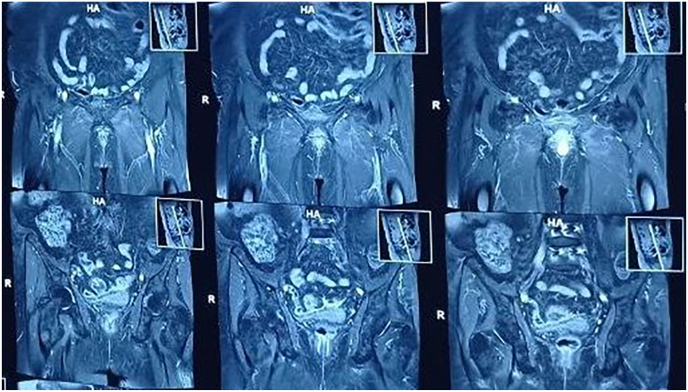
MRI showing that lesion protruding to anatomical location of the urethra.

**Fig. 4:**
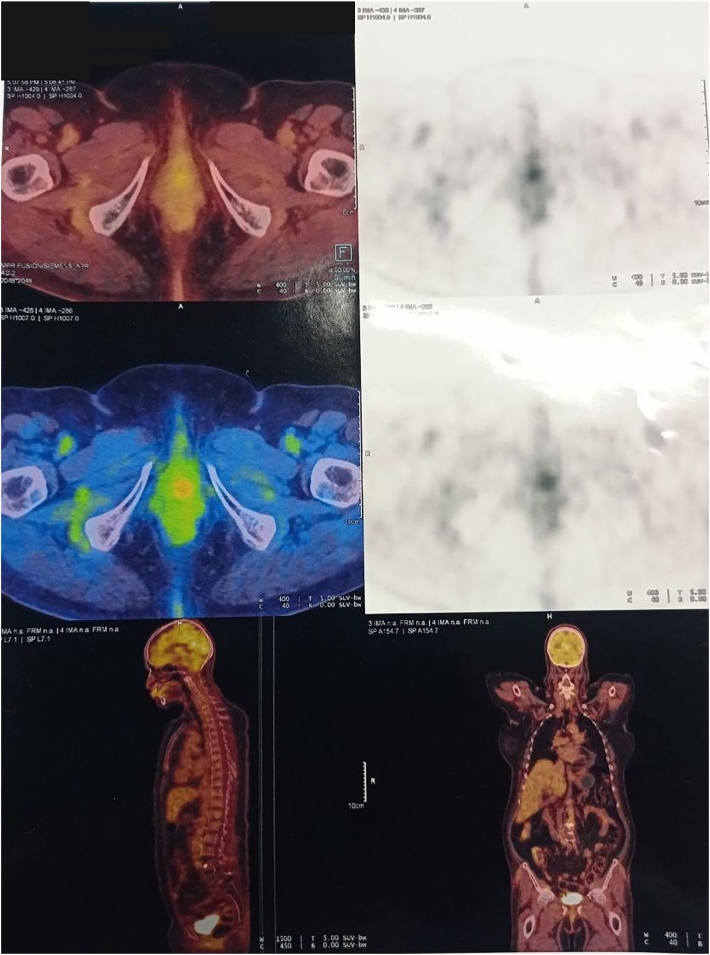
FDG PET-CT: FDG uptake in the distal part of the vagina.

Magnetic resonance imaging (MRI) of the pelvis revealed a 25 × 23 × 20 mm enhancing lesion at the anterior distal vaginal cuff, protruding into the anatomical location of the urethra. No adnexal masses were identified bilaterally. Chest, abdominal, and pelvic CT scans (CT Cystography) showed no evidence of metastasis or fistula formation. (Fig. 2, Fig. 3) 18F-FDG PET-CT demonstrated mild FDG uptake in the distal vagina, suggestive of the primary malignancy, with no additional hypermetabolic lesions noted (N0, M0) (Fig. 4).

Surgical management involved wide local excision of the urethral mass along with partial vaginectomy under spinal anesthesia. The Foley catheter was removed five days postoperatively, and the patient voided spontaneously without any signs of incontinence.

**Fig. 5:**
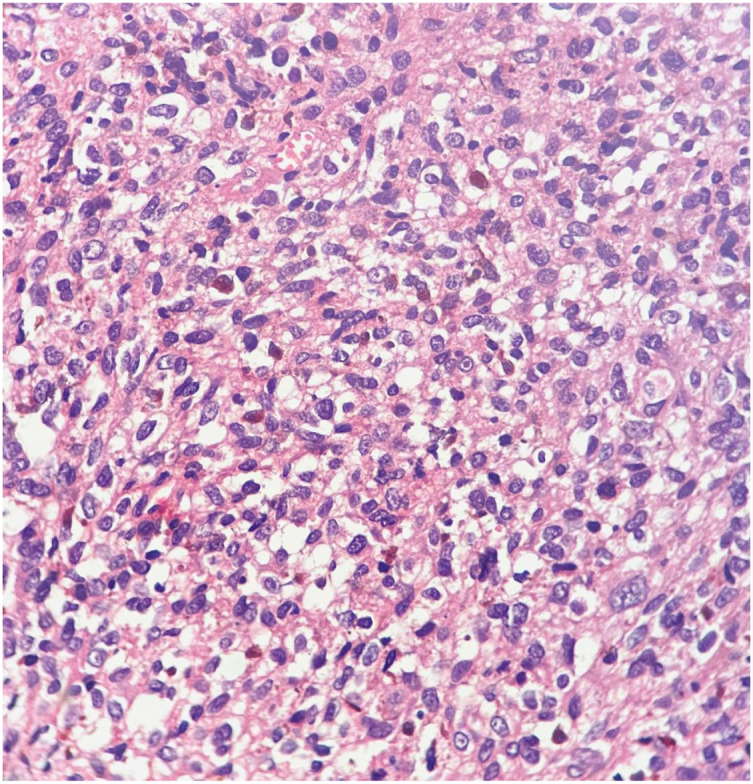
An irregular fragment of creamy brown soft to elastic tissue measuring 3 × 2.5 × 0.7cm is sent for pathology. Histopathologic examination revealed an ulcerated tissue diffusely infiltrated by oval to spindle cells with vesicular nuclei, occasional prominent eosinophilic nucleoli, moderate nuclear pleomorphism and eosinophilic cytoplasm with intracytoplasmic melanin pigment. Scattered mitotic figures are present (H&E stain, x400). The histopathologic findings are consistent with malignant melanoma.

Histopathological examination confirmed the diagnosis of malignant melanoma, characterized by ulcerated mucosa infiltrated with spindle and oval cells containing melanin pigment (Fig. 5). Immunohistochemical staining was positive for HMB-45 and Melan-A, supporting the diagnosis. Resection margins were not confirmed to be clear.

## 3 Follow-up

Six months following surgical resection, follow-up imaging revealed widespread metastatic disease. The patient was promptly referred to a medical oncologist, and systemic therapy—including immunotherapy—was initiated. Despite initial local control, the aggressive nature of urethral melanoma led to rapid disease progression, underscoring the importance of early diagnosis and the potential role of systemic treatments in improving long-term outcomes.

## 4 Discussion

Primary malignant melanoma of the urethra is an exceptionally rare entity, comprising less than 1 % of all melanomas and about 4 % of urethral cancers. It has a marked female predominance, with a reported female-to-male ratio of approximately 3:1, and most cases occur in individuals over the age of 60. The disease is more prevalent in Caucasian populations. Clinically, urethral melanoma often presents with nonspecific symptoms such as urethral bleeding, a palpable mass, hematuria, dysuria, or obstructive voiding. Due to its resemblance to more common benign lesions—particularly urethral caruncles—it is frequently misdiagnosed or diagnosed at a late stage. Notably, amelanotic variants, which account for up to 20 % of cases, further complicate clinical identification and require immunohistochemical confirmation for definitive diagnosis. The distal third of the female urethra is the most commonly affected site. Lesions often appear polypoid and may mimic benign conditions such as urethral polyps, mucosal prolapse, or caruncles. In some cases, pigmented caruncles can closely resemble melanotic melanomas, making histopathological analysis essential. Other differential diagnoses include transitional cell carcinoma and sarcoma of the urethra. Early and accurate diagnosis is crucial, given the high propensity for local recurrence and distant metastases. If a urethral mass exhibits suspicious features—such as rapid growth, firmness, ulceration, atypical pigmentation, or resistance to topical therapy—a prompt excisional biopsy should be performed to rule out malignancy. Delay in diagnosis is a major contributor to the poor prognosis commonly seen in these cases. Surgical excision remains the primary treatment modality. However, even with apparently complete resections, recurrence and metastasis are common. Recent advances in systemic therapy, particularly immune checkpoint inhibitors, offer hope for improved survival outcomes, although clinical experience in this specific subtype remains limited. This case highlights the diagnostic pitfalls of urethral melanoma and emphasizes the necessity of maintaining a high index of suspicion when evaluating atypical or persistent urethral lesions.^4^

## 5 Conclusion

This case underscores the importance of considering urethral melanoma in the differential diagnosis of urethral masses, (such as urethral caruncle) especially when the presentation mimics benign conditions. Early recognition, thorough staging, and prompt intervention are crucial for improving patient outcomes.

## CRediT authorship contribution statement

**Anahita Ansari Djafari:** Resources, Methodology, Investigation. **Mohammad Seifi Poor:** Writing – original draft, Supervision, Methodology, Investigation, Formal analysis, Data curation. **Sina Samenezhad:** Writing – review & editing. **Reza Nicknama:** Visualization. **Azadeh Rakhshan:** Data curation.
